# Tunneling Nanotube‐Mediated Transcellular Autophagy Alleviates Cadmium Induced Hepatocyte Injury

**DOI:** 10.1002/advs.202502793

**Published:** 2025-07-28

**Authors:** Tao Wang, Li Wang, Jian Sun, Yan Chen, Waseem Ali, Yonggang Ma, Ruilong Song, Xishuai Tong, Jiaqiao Zhu, Yan Yuan, Jianhong Gu, Jianchun Bian, Zongping Liu, Hui Zou

**Affiliations:** ^1^ College of Veterinary Medicine Yangzhou University Joint International Research Laboratory of Agriculture and Agri‐Product Safety of the Ministry of Education of China Institutes of Agricultural Science and Technology Development Yangzhou University Jiangsu Co‐innovation Center for Prevention and Control of Important Animal Infectious Diseases and Zoonoses Yangzhou 225009 China; ^2^ Jiangsu Key Laboratory of Zoonosis Yangzhou Jiangsu 225009 China

**Keywords:** autophagosome transfer, cadmium, TNFAIP2, transcellular autophagy, tunneling nanotubes

## Abstract

Cadmium (Cd) exposure is strongly linked to various diseases and dysregulation of autophagy is a pivotal mechanism in Cd toxicity. Targeted autophagy strategies are promising for the treatment of autophagy dysregulation‐related diseases, including Cd poisoning. However, the current understanding of autophagy mechanisms remains limited, hindering the development of effective strategies. Herein, a novel autophagy pathway, transcellular autophagy. Cd triggers this process in hepatocytes, facilitating the transfer of autophagosomes from damaged to healthy cells for degradation. Mechanistically, reactive oxygen species accumulation is a key driver of transcellular autophagy activation, while the disruption of autophagy fusion mechanisms triggers its activation. Notably, Cd‐induced transcellular autophagy relies on the tumor necrosis factor, alpha‐induced protein 2 (TNFAIP2)‐tunneling nanotube (TNT) system. Blocking this system prevents autophagosome transfer and exacerbates Cd‐induced autophagosome overload and apoptosis. The findings offer a novel perspective on autophagy, and provide new insights for targeted autophagy strategies to treat Cd poisoning and autophagy dysregulation‐related diseases.

## Graphical abstract

ROS accumulation is an important upstream signal for activating transcellular autophagy. Incomplete autophagy and TNT formation are necessary conditions for activating transcellular autophagy, and the disruption of the autophagic fusion mechanism is a trigger for activating transcellular autophagy. Cd activates transcellular autophagy and promotes the transfer of autophagosomes from Cd‐damaged cells to healthy ones for fusion and degradation.

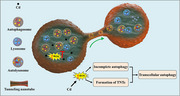

## 1 Introduction

Pollution is a leading environmental risk factor for disease and premature mortality worldwide.^[^ ^1^ ^]^ Cadmium (Cd) is a significant environmental pollutant that poses a serious threat to human and animal health. Cd accumulates in multiple organs throughout the food chain and is closely related to a range of diseases, including neurodegenerative, bone, liver, and kidney diseases, and various types of cancer.^[^ ^2^, ^3^, ^4^, ^5^, ^6^ ^]^ Notably, the liver accounts for ≈30% of Cd accumulation in the body, making it one of the organs most severely affected by Cd toxicity.^[^ ^7^ ^]^ However, an incomplete understanding of the mechanisms underlying Cd toxicity remains the primary hurdle in the development of targeted protective products.

Autophagy is a cellular quality control mechanism that targets various intracellular components for degradation, and plays a crucial role in promoting cell survival and restoring cellular homeostasis.^[^ ^8^, ^9^ ^]^ Given the significance of autophagy in disease progression, targeting it is considered a promising and effective treatment approach with immense potential.^[^ ^10^, ^11^ ^]^ Selective autophagy inhibitors targeting the autophagy process have been developed for cancer treatment.^[^ ^12^ ^]^ For instance, the inhibitors, chloroquine and hydroxychloroquine, which act at the autophagy fusion stage, have undergone clinical trials for cancer treatment.^[^ ^13^, ^14^ ^]^ These inhibitors enhance the sensitivity of cancer cells to chemotherapeutic drugs, thereby potentiating their therapeutic effects. Notably, autophagy dysregulation is an important cytotoxic mechanism of Cd.^[^ ^15^ ^]^ Targeting autophagy also has substantial therapeutic potential for the prevention and treatment of Cd poisoning. Cd induces incomplete autophagy by blocking autophagic flux, ultimately leading to cell damage.^[^ ^16^, ^17^, ^18^, ^19^ ^]^ Based on this mechanism, several drugs, such as puerarin, taurine, quercetin, melatonin, paeonol, and trehalose alleviate Cd‐induced cell damage by restoring autophagic flux.^[^ ^20^, ^21^, ^22^, ^23^, ^24^, ^25^, ^26^ ^]^ Therefore, targeting the regulation of autophagic flux is currently the main research direction of targeted autophagy strategies for the prevention and treatment of Cd poisoning. However, the current understanding of autophagy mechanisms remains limited, thereby hampering the further development of effective targeted autophagy strategies for the treatment of diseases associated with autophagy dysregulation.

Multicellular organisms maintain intercellular communication through various mechanisms that are essential for cellular and tissue homeostasis.^[^ ^27^ ^]^ Cells located far apart can exchange signals and substances via secreted signaling molecules or exosomes, whereas adjacent cells can communicate directly through gap junctions.^[^ ^28^, ^29^, ^30^ ^]^ Our previous study revealed that Cd‐exposed hepatocytes could induce bystander effects via the transmission of substances or signals between cells through gap junction intercellular communication.^[^ ^31^ ^]^ Nevertheless, gap junction channels are inherently limited in length, making it challenging to observe substances transferred within these channels using current experimental techniques. Tunneling nanotubes (TNTs) represent a novel form of direct intercellular communication capable of achieving long‐distance direct communication between cells.^[^ ^32^ ^]^ TNTs offer a distinctive open membrane channel for intercellular substance exchange, with diameters spanning from tens to hundreds of nm and lengths extending up to 10–100 µm or longer.^[^ ^32^, ^33^ ^]^ TNTs enable intercellular transfer of substances, including small molecules, protein aggregates, various organelles, and pathogens.^[^ ^34^, ^35^, ^36^ ^]^ The transfer of organelles between cells plays an important role in maintaining cellular homeostasis, regulating cellular functions, and responding to external stimuli.^[^ ^37^, ^38^ ^]^ Mitochondria are the main organelles responsible for ATP production, and mitochondrial quality control is crucial for maintaining mitochondrial function and cellular homeostasis.^[^ ^39^ ^]^ Recently, mitochondrial transfer, a form of intercellular signaling, is widely studied. Intercellular mitochondrial transfer plays an important role in mitochondrial quality control and maintaining tissue homeostasis.^[^ ^40^ ^]^ Our recent study reported the role of mitochondrial transfer in Cd‐induced nonalcoholic fatty liver disease.^[^ ^41^ ^]^ Unexpectedly, during the research process, we found that autophagosomes can also transfer between hepatocytes. Notably, intercellular autophagosome transfer occurs both between cells of the same type, such as human squamous cell carcinoma cells, and cells of different types, such as from B‐cell precursor acute lymphoblastic leukemia cells to mesenchymal stromal cells (MSCs).^[^ ^42^, ^43^ ^]^ However, there are currently very limited reports on autophagosome transfer. Existing studies primarily focus on reporting the phenomenon, while the underlying mechanisms and functional significance of this process remain elusive. Notably, a recent study reported the transfer of lysosomes from healthy podocytes to advanced glycation end product (AGE)‐damaged podocytes, whereas autophagosomes were transferred from AGE‐damaged to healthy podocytes.^[^ ^37^ ^]^ This suggests that there is a potential for transcellular degradation of autophagosomes, which could represent a novel protective mechanism to alleviate the pressure caused by autophagosome overload.

The specific pathways and signaling mechanisms involved in the transcellular degradation of autophagosomes in hepatocytes, particularly in the context of Cd exposure, remain unclear. In this study, we describe a novel autophagy pathway, transcellular autophagy. Our findings reveal that Cd triggers transcellular autophagy by disrupting autophagy fusion mechanisms, with reactive oxygen species (ROS) serving as a crucial upstream signal. This process involves the transfer of autophagosomes from Cd‐injured hepatocytes to adjacent healthy hepatocytes in a TNT‐dependent manner, thereby alleviating autophagosome accumulation in Cd‐injured hepatocytes. These findings reveal a unique protective mechanism that can alleviate the accumulation of autophagosomes mediated by incomplete autophagy and provide a new direction for the study of autophagy‐targeting strategies in disease intervention.

## 2 Results

### 2.1 Transcellular Autophagy

**Figure 1:**
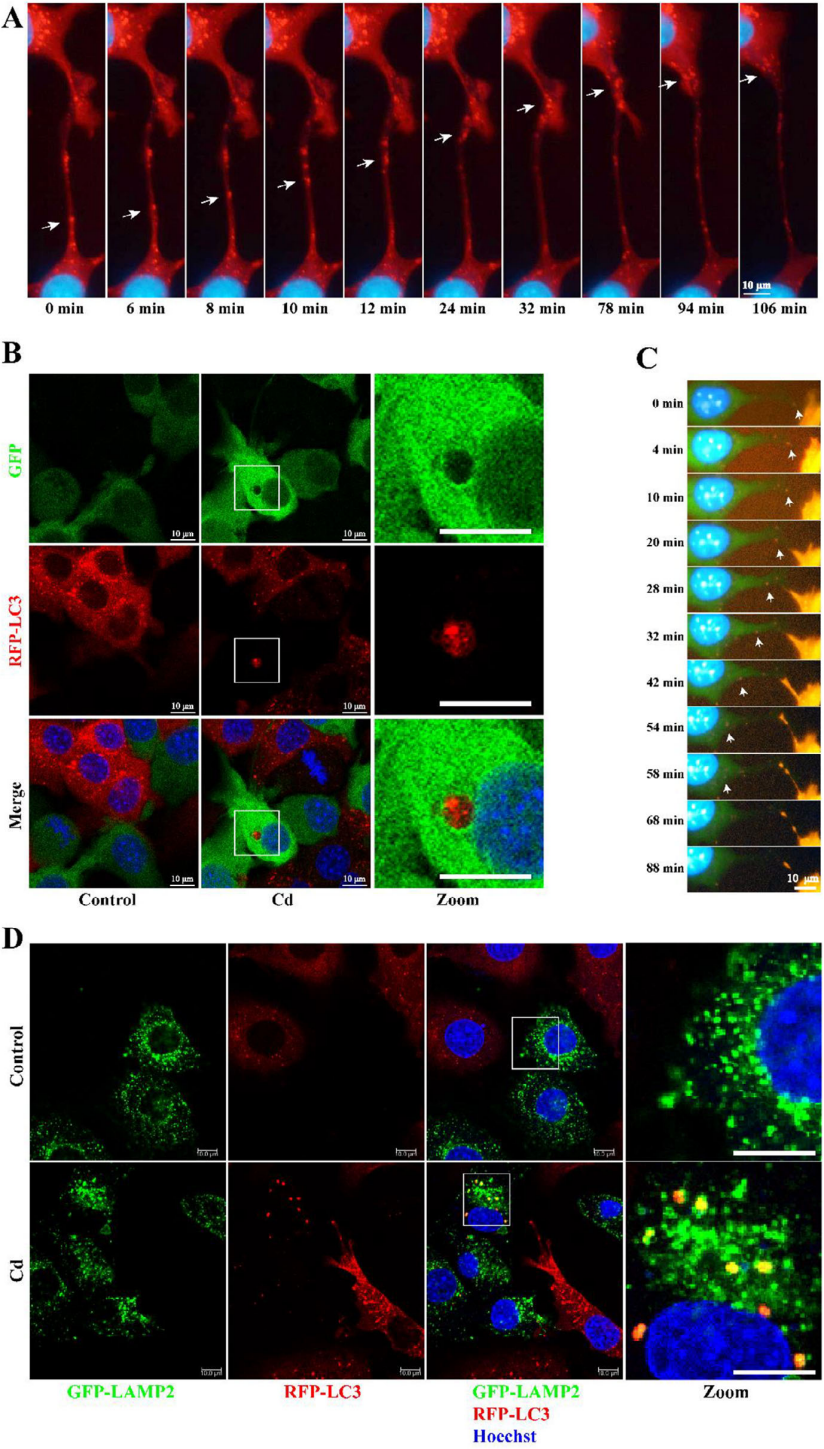
Transcellular autophagy. AML12 cells labeled with RFP‐LC3 were exposed to 5 µm Cd and imaged every 2 min using a high‐content analysis system. A) Live cell imaging of the autophagosomes transfer process. Scale bar, 10 µm. AML12 cells labeled with RFP‐LC3 were co‐cultured with AML12 cells labeled with GFP at a ratio of 1:1 and treated with 5 µm Cd for 6 h. B) Confocal microscopy images of LC3 puncta distribution. Scale bar, 10 µm. C) Live cell imaging of the degradation process of autophagosomes transferred to adjacent cells. Scale bar, 10 µm. AML12 cells labeled with RFP‐LC3 were co‐cultured with AML12 cells labeled with GFP‐LAMP2 at a ratio of 1:1 and treated with 5 µm Cd for 6 h. D) Confocal microscopy images of co‐localization of LC3 with LAMP2. Scale bar, 10 µm.

To investigate transcellular autophagy, we labeled autophagosomes with expressing red fluorescence protein‐microtubule associated protein 1 light chain 3 alpha [LC3] (RFP‐LC3) and observed the dynamic process of LC3 puncta transfer between AML12 cells using a high‐content analysis system. **Figure** **1**A and Video S1 (Supporting Information) show that LC3 puncta were gradually transferred from one cell to another through the tubular structure between cells. In the co‐culture model of AML12 cells labeled with RFP‐LC3 or green fluorescent protein (GFP) we found a distribution of RFP‐LC3 puncta in GFP‐labeled cells in the Cd group (Figure 1B), indicating that Cd can induce autophagosome transfer between AML12 cells. Subsequently, in the co‐culture model of AML12 cells labeled with RFP‐LC3 or GFP, LC3 puncta gradually disappeared after transferring to adjacent cells (Figure 1C; Video S2, Supporting Information). This indicates that Cd can induce autophagosome transfer to adjacent cells for degradation. To verify whether autophagosomes transferred to adjacent cells could fuse with lysosomes in adjacent cells, we established co‐culture models of AML12 cells labeled with RFP‐LC3 or GFP‐LAMP2 (lysosomal associated membrane protein 2; lysosome tags). As shown in Figure 1D, LC3 and LAMP2 in GFP‐LAMP2 labeled cells in the Cd group. This indicates that Cd can induce autophagosome transfer to adjacent cells and fusion with lysosomes in adjacent cells. These results indicate that autophagosomes can be transferred from one cell to a neighboring cell, where they fuse with lysosomes for degradation. We refer to this phenomenon as transcellular autophagy.

### 2.2 Cd activates Transcellular Autophagy and Promotes the Transfer of Autophagosomes from Damaged Cells to Normal Cells

**Figure 2:**
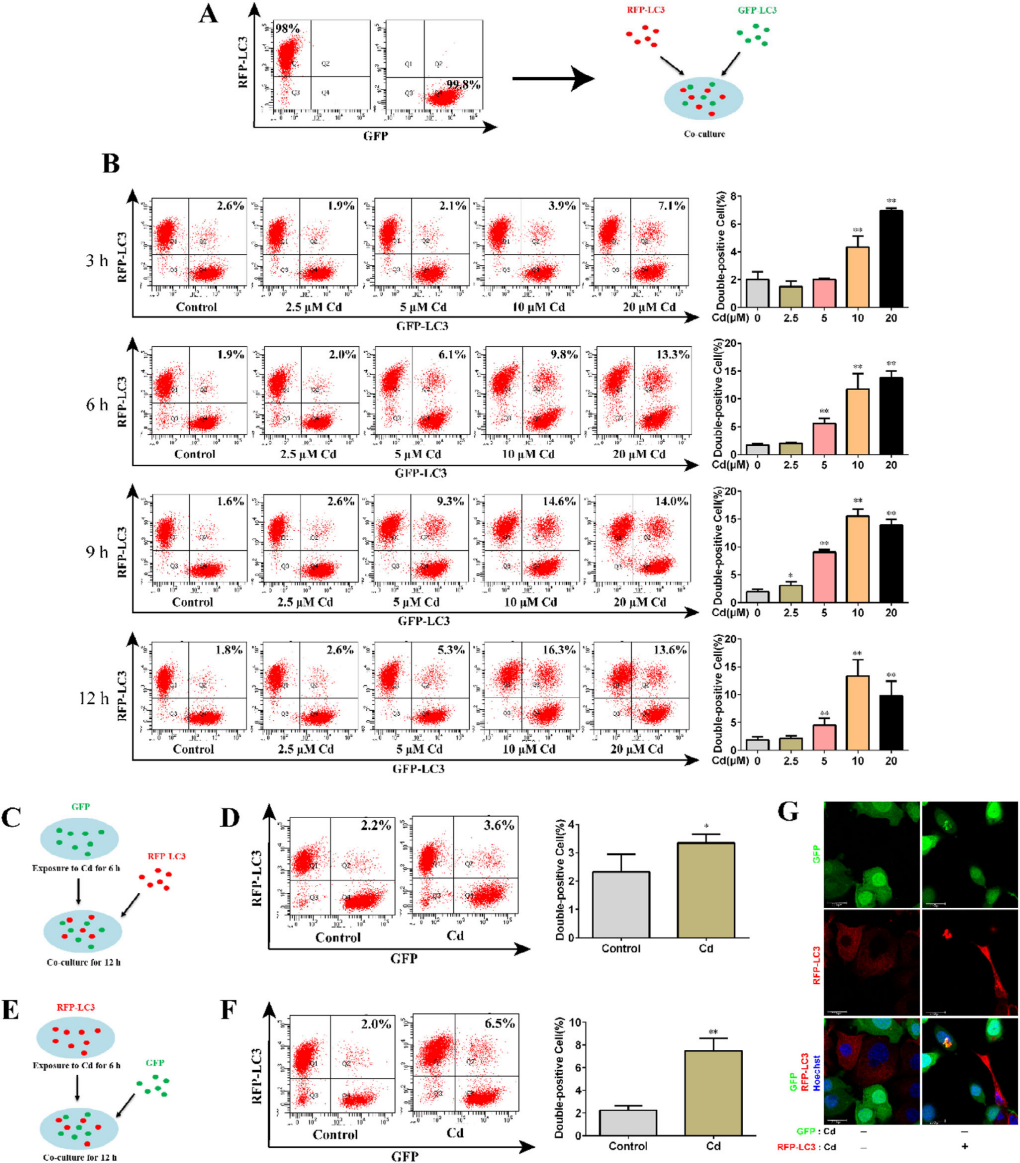
Effect of Cd on transcellular autophagy in AML12 cells. A) Schematic diagram of the co‐culture model of AML12 cells labeled with RFP‐LC3 and AML12 cells labeled with GFP‐LC3. B) In the co‐culture model, different concentrations of Cd were exposed for different times, and the LC3 transfer rate was analyzed using flow cytometry (*n* = 3). C) Schematic diagram of the model for evaluating the transfer of autophagosomes from normal to damaged cells. D) Flow cytometry analysis of the change in the transfer rate of LC3 from normal to damaged cells (*n* = 3). E) Schematic diagram of the model for evaluating the transfer of autophagosomes from damaged to normal cells. F) Flow cytometry analysis of the change in the transfer rate of LC3 from damaged to normal cells (*n* = 3). G) Representative confocal images of the transfer of autophagosomes from damaged to normal cells. Scale bar, 20 µm. Data are expressed as the mean ± SD. Statistical analysis was conducted using one‐way analysis of variance and Scheffe's F test. Compared with the control group, ^*^ *p* < 0.05, ^**^ *p* < 0.01.

To evaluate the effect of Cd on transcellular autophagy in AML12 cells, we mixed RFP‐LC3‐labeled cells with GFP‐LC3‐labeled cells at a 1:1 ratio to establish a co‐culture model (**Figure** **2**A). Changes in the double‐positive fluorescence rate were analyzed using flow cytometry to reflect changes in the LC3 transfer rate, which was used to quantitatively assess transcellular autophagy. Figure 2B shows that the changes in the double‐positive fluorescence rate exhibited the same trend at different time points of Cd exposure; as the Cd exposure dose increased, the double‐positive fluorescence rate gradually increased. This indicates that Cd activates transcellular autophagy in AML12 cells. Based on the above results, 10 µm Cd exposure for 6 h was selected for subsequent mechanism research, as under this exposure condition, transcellular autophagy was ongoing and maintained at a relatively high level.

To investigate the directionality of autophagosome transfer between AML12 cells, we established two co‐culture models. In the co‐culture model evaluating the transfer of autophagosomes from normal to damaged cells (Figure 2C), we observed a slight increase in the fluorescence double‐positive rate in the Cd group compared with that in the control group (*p*< 0.05), ≈1.6 times higher than that in the control group (Figure 2D). However, when examining the transfer of autophagosomes from damaged cells to normal cells in the co‐culture model (Figure 2E), the fluorescence double‐positive rate in the Cd group significantly increased (*p*< 0.01), ≈3.2 times higher than that in the control group (Figure 2F), which was much higher than the change in the transfer rate of autophagosomes from normal to damaged cells. fluorescent observations are presented in Figure 2G, depicting the transfer of autophagosomes from damaged (labeled with RFP‐LC3) to adjacent normal cells (labeled with GFP). These findings suggest that the transfer of autophagosomes between cells is bidirectional, and during Cd‐induced transcellular autophagy, autophagosomes are primarily transferred from damaged to normal cells.

### 2.3 Transcellular Autophagy Requires TNTs

To investigate the primary pathways of autophagosome transfer between AML12 cells, we examined the role of extracellular vesicles/endocytosis, gap junctions, and TNTs in Cd‐induced transcellular autophagy in AML12 cells. Using a Transwell co‐culture model and gap junction inhibitors (Gap 27 and 18‐β‐glycyrrhetinic acid [GA]), we excluded the dependence of intercellular autophagosome transfer on extracellular vesicles/endocytosis and gap junctions (Figure S1, Supporting Information).

**Figure 3:**
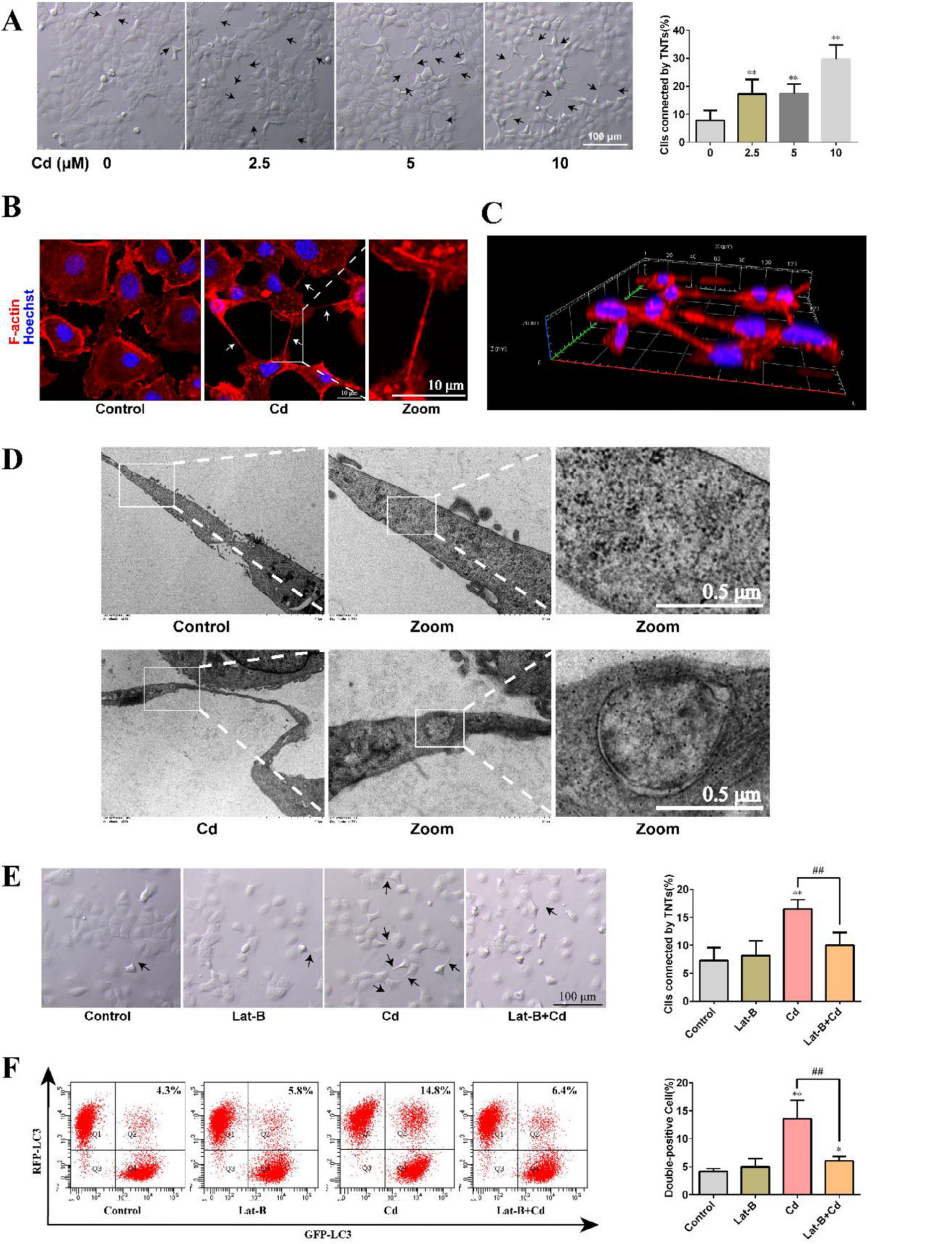
Cd induced transcellular autophagy requires TNTs. AML12 cells were exposed to various concentrations of Cd for 6 h. A) TNT‐like structures between cells under brightfield microscopy (TNTs were highlighted with black arrows). Scale bar, 100 µm (*n* = 6). AML12 cells was exposed to 10 µm Cd for 6 h. B) Phalloidin staining was used to characterize TNTs (TNTs were highlighted with white arrows). Scale bar, 10 µm. C) Confocal 3D imaging of TNTs. D) TEM images of autophagosomes in TNTs. Scale bar, 0.5 µm. Cells were pretreated with 100 nm Lat‐B for 2 h and then treated with 10 µm Cd for 6 h. E) changes in the number of TNTs (TNTs were highlighted with black arrows). Scale bar, 100 µm (*n* = 6). F) Flow cytometry of LC3 transfer rate (*n* = 3). Data are expressed as the mean ± SD. Statistical analysis was conducted using one‐way analysis of variance and Scheffe's F test. Compared with the control group, ^*^ *p* < 0.05, ^**^ *p* < 0.01. Compared with the Cd group, # *p* < 0.05, ## *p* < 0.01.

To further investigate the role of TNTs in Cd‐induced transcellular autophagy, we examined the effects of Cd on TNT formation in AML12 cells. As shown in **Figure** **3**A, Cd exposure promoted the formation of TNT‐like structures in AML12 cells (*P*< 0.01). Subsequently, we characterized TNTs using phalloidin staining. As shown in Figure 3B,C, the intercellular connecting structures in the Cd group were primarily composed of F‐actin and 3D imaging revealed tubular structures connecting the two cells, which is consistent with the reported structure of TNTs.^[^ ^32^ ^]^ These data indicate that Cd promotes the formation of TNTs. Notably, transmission electron microscopy (TEM) revealed the presence of autophagosomes within TNTs (Figure 3D). Next, we investigated the effects of TNT inhibition on Cd‐induced transcellular autophagy. As shown in Figure 3E,F, inhibition of TNT formation using the TNT inhibitor, Latrunculin B (Lat‐B), suppressed Cd‐induced transcellular autophagy. Collectively, these findings suggest that Cd‐induced transcellular autophagy is dependent on TNTs.

### 2.4 RNA Sequencing (RNA‐seq) of Potential Upstream Signals Regulating Cd‐Induced Transcellular Autophagy

**Figure 4:**
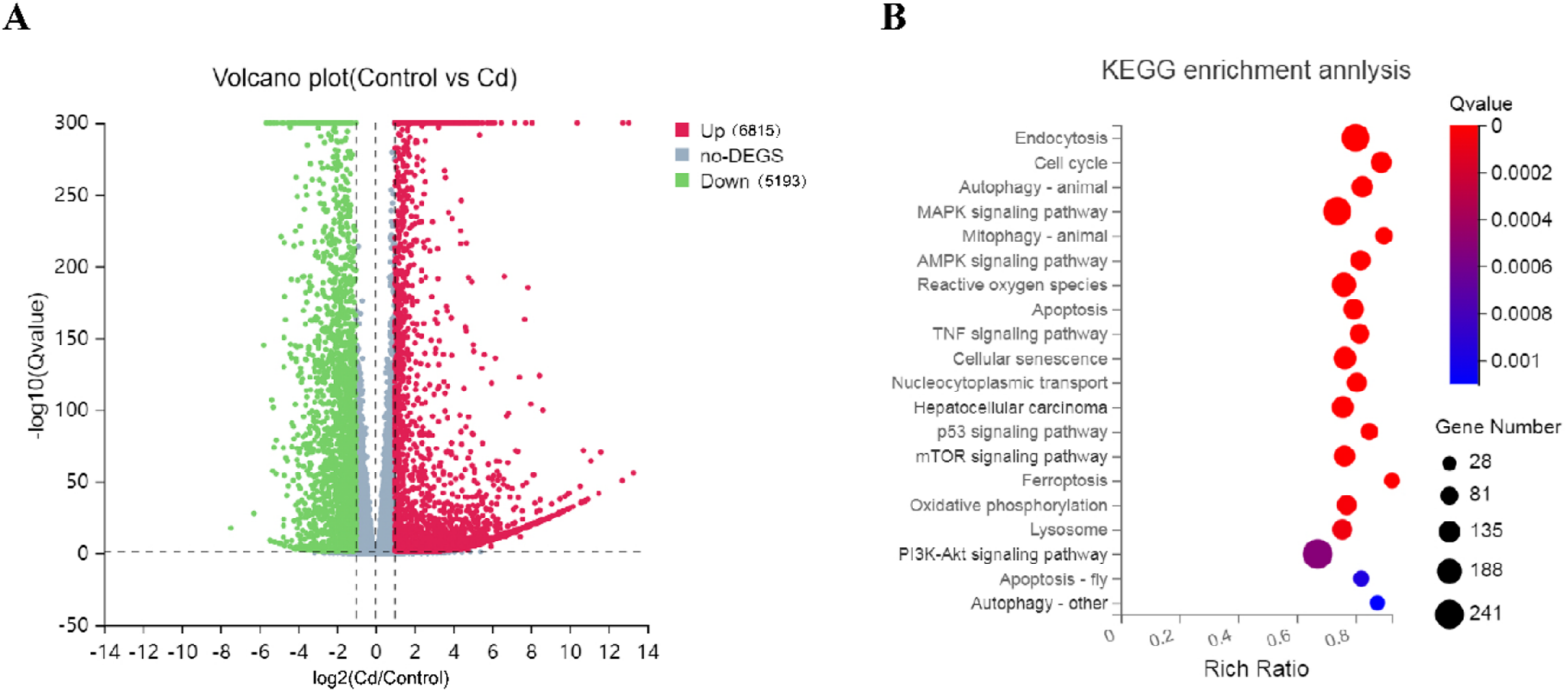
RNA‐seq analysis to investigate potential upstream signals regulating Cd‐induced transcellular autophagy. AML12 cells were treated with 10 µm Cd for 6 h, followed using RNA‐seq analysis (*n* = 6). A) Volcano plot of DEGs. B) KEGG pathway enrichment analysis.

To investigate the signaling pathways upstream of Cd‐induced transcellular autophagy, we performed RNA‐seq analysis. As shown in **Figure** **4**A, 12008 differentially expressed genes (DEGs) were identified between the Control and Cd groups, of which 6815 were upregulated and 5193 were downregulated. KEGG enrichment analysis revealed that the DEGs were primarily enriched in endocytosis, the cell cycle, autophagy, MAPK signaling pathway, Mitophagy, AMPK signaling pathway, ROS, apoptosis, and other related pathways (Figure 4B). Given that autophagy is a fundamental prerequisite for cell‐to‐cell transmission of autophagic components and that ROS act as crucial upstream modulators of the aforementioned signaling pathways, this study focused on autophagy and ROS signaling.

### 2.5 Incomplete Autophagy is Required for the Induction of Transcellular Autophagy

**Figure 5:**
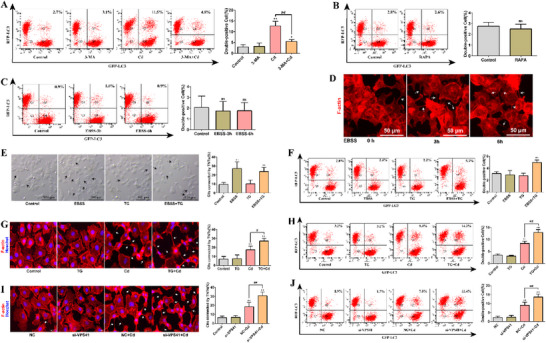
Incomplete autophagy is required for the induction of transcellular autophagy. AML12 cells were pretreated with 5 mm 3‐MA for 1 h and then treated with 10 µm Cd for 6 h. A) Flow cytometry analysis of LC3 transfer rate (*n* = 3). AML12 cells were treated with 100 nm RAPA for 6 h. B) Flow cytometry analysis of LC3 transfer rate (*n* = 3). Cells were treated with EBSS for 3 h or 6 h. C) Flow cytometry analysis of LC3 transfer rate (*n* = 3). D) Observation of TNTs using Phalloidin staining. AML12 cells were treated with EBSS and 50 nm TG for 3 h. E) Observation of TNT formation (TNTs were highlighted with black arrows). Scale bar, 100 µm. F) Flow cytometry analysis of LC3 transfer rate (*n* = 3). AML12 cells were treated with 50 nm TG and 10 µm Cd for 6 h. G) Observation of TNT formation (TNTs were highlighted with white arrows). Scale bar, 10 µm. H) Flow cytometry analysis of LC3 transfer rate (*n* = 3). After silencing VPS41, AML12 cells were treated with 10 µm Cd for 6 h. I) Observation of TNT formation (TNTs were highlighted with white arrows). Scale bar, 10 µm. J) Flow cytometry analysis of LC3 transfer rate (*n* = 3). Data are expressed as the mean ± SD. Statistical analysis was performed using one‐way analysis of variance and Scheffe's F test. Compared with the control group, ^*^ *p* < 0.05, ^**^ *p* < 0.01. Compared with the Cd or NC+Cd group, ^#^ *p* < 0.05, ^##^ *p* < 0.01.

First, we investigated the role of autophagy activation signals in Cd‐induced transcellular autophagy. We treated the cells with 3‐Methyladenine (3‐MA; an inhibitor of autophagy) in combination with Cd and analyzed transcellular autophagy using flow cytometry. As expected, inhibition of autophagy by 3‐MA significantly reduced Cd‐induced transcellular autophagy (**Figure** **5**A). We obtained the same result with AMPK pathway (important for autophagy regulation) inhibition (Figure S2, Supporting Information). This indicated that autophagy activation is a prerequisite for Cd‐induced transcellular autophagy. Subsequently, we investigated whether autophagy activation could directly activate transcellular autophagy. Autophagy was activated using rapamycin (RAPA; an autophagy activator) and transcellular autophagy was analyzed using flow cytometry. Notably, RAPA‐induced autophagy did not directly activate transcellular autophagy (Figure 5B). We validated this finding by activating autophagy during starvation. Our previous study showed that the initiation and termination of starvation‐induced autophagy are dynamic, with autophagy activated after 3 h of starvation and terminated after 6 h of starvation.^[^ ^44^ ^]^ However, this study showed that starvation for 3 or 6 h did not directly activate transcellular autophagy (Figure 5C). Given that transcellular autophagy mainly depends on TNTs, we evaluated the effect of starvation on the formation of TNTs using phalloidin staining. Notably, the results showed that starvation for 3 or 6 h promoted the formation of TNTs (Figure 5D). This indicates that starvation can activate autophagy and promote the formation of TNTs but cannot directly activate transcellular autophagy. Collectively, these data suggest that activation of autophagy is a prerequisite for Cd‐induced transcellular autophagy, but not a direct trigger.

We further investigated the effect of disruption of the autophagy fusion mechanism on transcellular autophagy. First, we analyzed the changes in TNT formation and transcellular autophagy under starvation and Thapsigargin (TG; an inhibitor of autophagosome‐lysosome fusion) treatment. As shown in Figure 5E,F, Earle's balanced salt solution (EBSS) treatment alone induced TNT formation but did not activate transcellular autophagy, whereas TG treatment alone did not induce TNT formation or activate transcellular autophagy. Combined treatment with EBSS and TG induced TNT formation and activated transcellular autophagy. This indicated that blocking the fusion of autophagosomes and lysosomes can activate transcellular autophagy by activating autophagy and inducing TNT formation. Second, we investigated the effect of disrupting the autophagy fusion mechanism on Cd‐induced transcellular autophagy using combined treatment with Cd and TG. As shown in Figure 5G,H, the inhibition of autophagosome‐lysosome fusion further promoted Cd‐induced TNT formation and transcellular autophagy. Our previous study showed that silencing vacuolar protein sorting 41 (VPS41) can inhibit autophagosome‐lysosome fusion, thereby aggravating Cd‐induced incomplete autophagy.^[^ ^44^ ^]^ We further verified the role of the disruption of the autophagy fusion mechanism in Cd‐induced transcellular autophagy by silencing VPS41 to inhibit autophagosome‐lysosome fusion. As shown in Figure 5I,J, silencing of VPS41 further promoted Cd‐induced TNT formation and transcellular autophagy. These data suggest that disruption of the autophagy fusion mechanism triggers Cd‐induced transcellular autophagy. Collectively, these results indicated that incomplete autophagy was required for the induction of transcellular autophagy.

### 2.6 Accumulation of ROS is a Driving Factor for Activating Transcellular Autophagy

**Figure 6:**
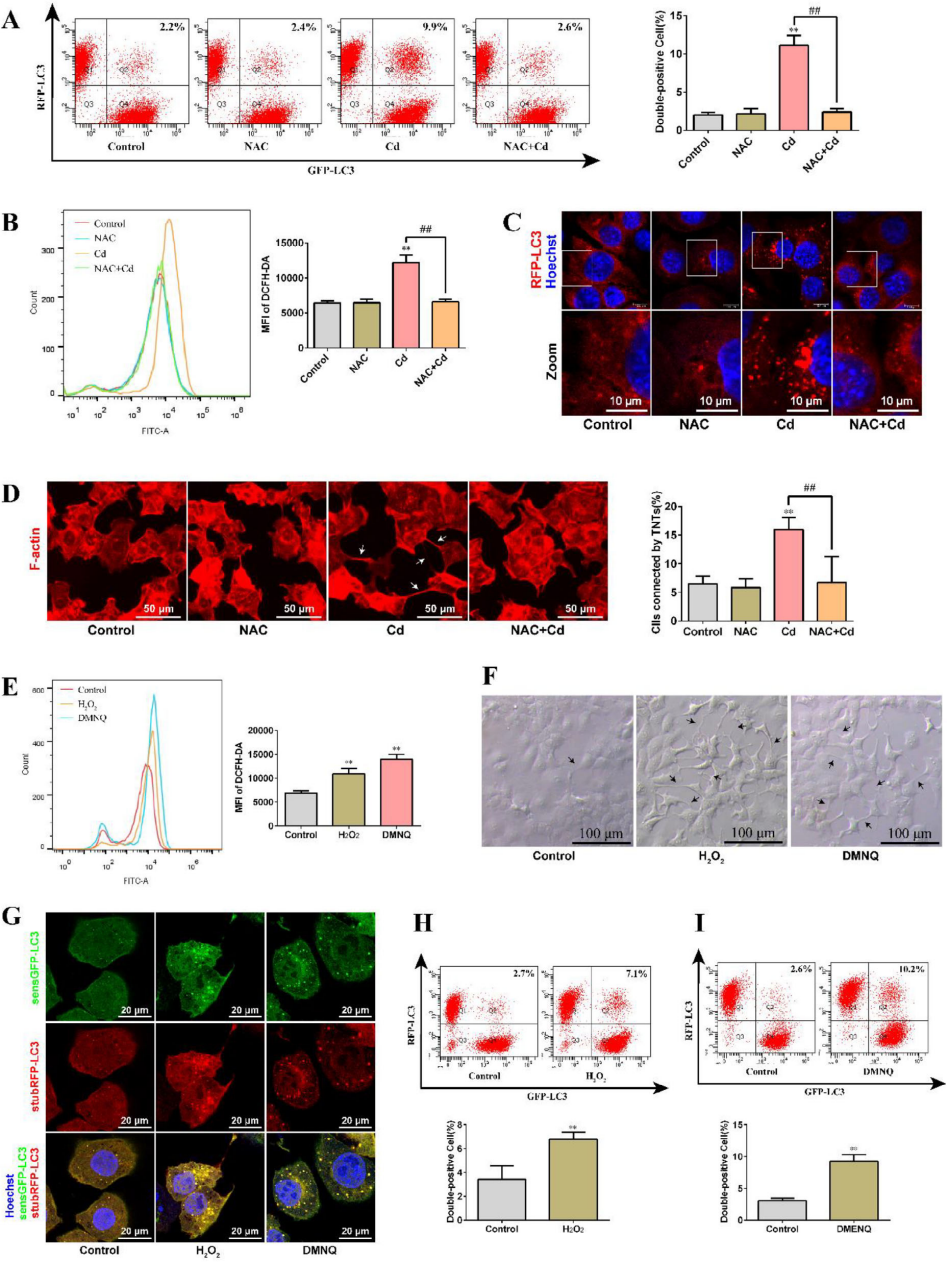
The accumulation of ROS is a driving factor for activating transcellular autophagy. AML12 cells were pretreated with 400 µm NAC for 1 h and then treated with 10 µm Cd for 6 h. A) Flow cytometry analysis of LC3 transfer rate (*n* = 6). B) Flow cytometry analysis of ROS levels (*n* = 6). C) Confocal microscopy images of RFP‐LC3 puncta. Scale bar, 10 µm. D) Phalloidin staining to observe TNTs (TNTs were highlighted with white arrows). Scale bar, 50 µm (*n* = 4). AML12 cells were treated with 400 nm H_2_O_2_ or 100 µm DMNQ for 6 h. E) Flow cytometry analysis of ROS levels (*n* = 3). F) Brightfield microscopy observation of TNTs (TNTs were highlighted with black arrows). Scale bar, 100 µm. G) RFP‐GFP‐LC3 fluorescence analysis of autophagic flux. Scale bar, 20 µm. H, I) Flow cytometry analysis of LC3 transfer rate (*n* = 3). Data are expressed as the mean ± SD. Statistical analysis was performed using one‐way analysis of variance and Scheffe's F test. Compared with the control group, ^*^ *p* < 0.05, ^**^ *p* < 0.01. Compared with the Cd group, ^#^ *p* < 0.05, ^##^ *p* < 0.01.

To investigate the role of ROS in Cd‐induced transcellular autophagy in AML12 cells, we pretreated cells with 400 µm N‐acetyl‐L‐cysteine (NAC; a ROS scavenger) for 1 h followed by combined treatment with 5 µm Cd for 6 h. Changes in ROS levels and transcellular autophagy were analyzed using flow cytometry. As shown in **Figure** **6**A,B, the Cd‐induced accumulation of ROS was completely inhibited by NAC, which also completely inhibited Cd‐induced transcellular autophagy. Subsequently, we analyzed changes in autophagy levels and TNT formation. As shown in Figure 6C,D, NAC inhibited Cd‐induced autophagy activation and TNT formation. These results suggest that ROS scavenging inhibits both autophagy activation and TNT formation, ultimately suppressing Cd‐induced transcellular autophagy. Therefore, ROS accumulation is an important upstream signal for Cd‐induced transcellular autophagy.

To further confirm that the accumulation of ROS is an important upstream signal for activating transcellular autophagy, cells were treated with 400 nm H_2_O_2_ or 100 µm 2,3‐dimethoxy‐1,4‐naphthoquinone (DMNQ; a ROS inducer) for 6 h, and then the levels of ROS, TNT formation, autophagic flux, and changes in transcellular autophagy were analyzed. As expected, H_2_O_2_ and DMNQ induced ROS accumulation (Figure 6E), promoted TNT formation (Figure 6F), blocked autophagic flux (Figure 6G), and ultimately activated transcellular autophagy (Figure 6H,I). These data further confirm that ROS accumulation is an important upstream signal that induces transcellular autophagy.

### 2.7 Cd‐induced Transcellular Autophagy Depends on the TNFAIP2‐TNT System

**Figure 7:**
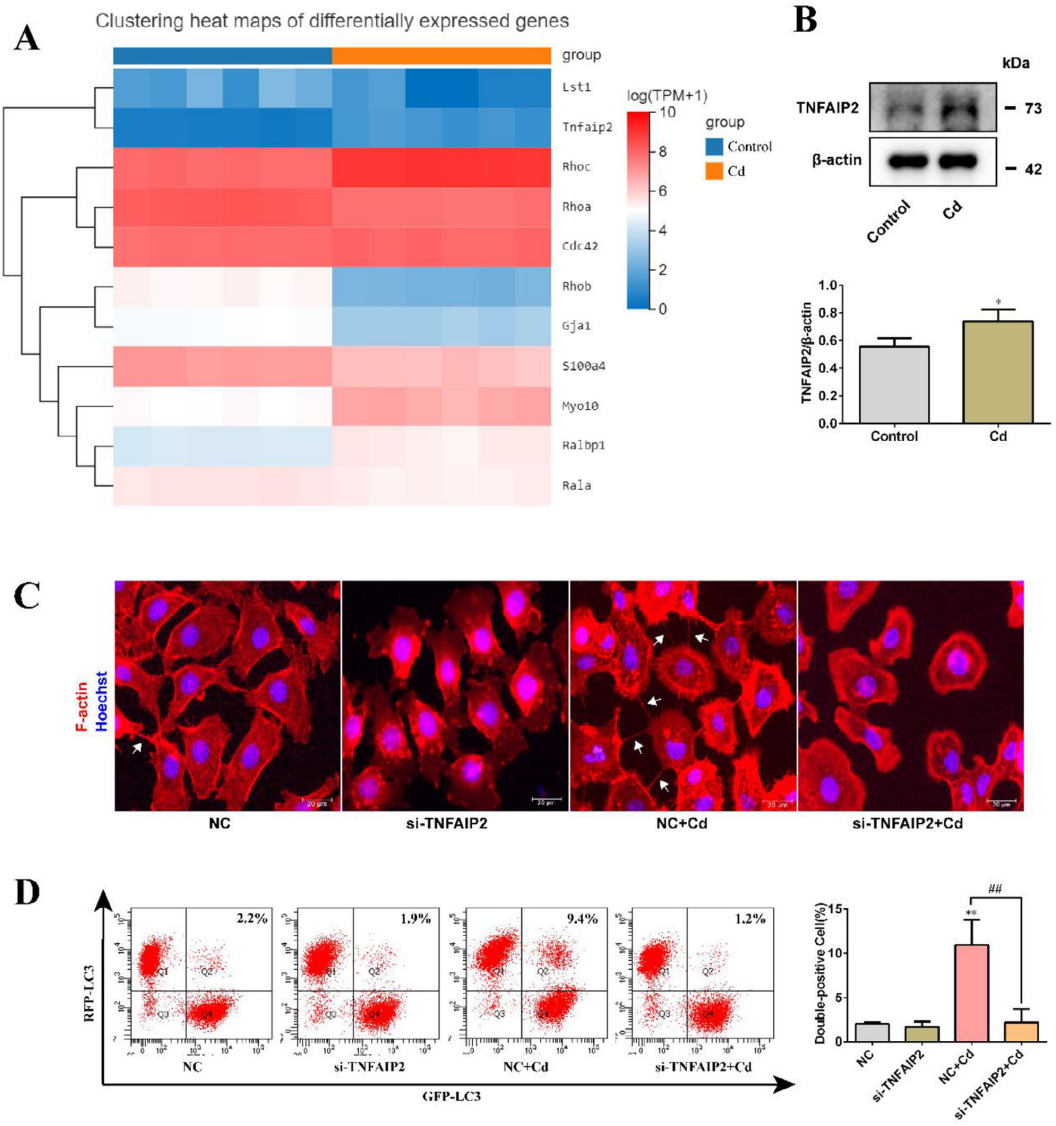
Cd‐induced transcellular autophagy depends on the TNFAIP2‐TNT system. AML12 cells were treated with 10 µm Cd for 6 h. A) RNA‐seq analysis of gene expression related to TNT formation (*n* = 6). B) WB analysis of TNFAIP2 protein expression level (*n* = 3). After silencing TNFAIP2, AML12 cells were treated with 10 µm Cd for 6 h. C) Phalloidin staining analysis of TNT formation (TNTs were highlighted with white arrows). Scale bar, 20 µm. D) Flow cytometry analysis of LC3 transfer rate (*n* = 3). Data are expressed as the mean ± SD. Statistical analysis was performed using one‐way analysis of variance and Scheffe's F test. Compared with the control group, ^*^ *p* < 0.05, ^**^ *p* < 0.01. Compared with the NC+Cd group, ^#^ *p* < 0.05, ^##^ *p* < 0.01.

To identify the key proteins that regulate TNT formation in AML12 cells, we first analyzed the expression of genes related to TNT formation using RNA‐seq. As shown in **Figure** **7**A, Cd exposure led to the upregulation of genes, such as *Tnfaip2*, *Rhoc*, *Cdc42*, *Myo10*, and *Ralbp1*, whereas genes, such as *Rhoa*, *Rhob*, *Gja1*, *S100a4*, and *Rala* were downregulated. *Lst1* showed no significant changes. After silencing *Rhoc*, *Cdc42*, *Myo10*, *Ralbp1*, and *Lst1* using small‐interfering RNA (siRNA), cells were treated with 5 µm Cd for 6 h to analyze the formation of TNTs. As shown in Figures S3 and S4 (Supporting Information), the silencing of *Rhoc*, *Cdc42*, *Myo10*, *Ralbp1*, and *Lst1* did not inhibit the formation of TNTs induced by Cd. We investigated the role of TNFAIP2 in Cd‐induced TNT formation. Western blotting (WB) results showed that Cd promoted the expression of the TNFAIP2 protein (Figure 7B). Phalloidin staining revealed that TNFAIP2 silencing inhibited Cd‐induced TNT formation (Figure 7C). This suggests that TNFAIP2 is a key protein involved in Cd‐induced TNT formation in AML12 cells. We investigated the role of TNFAIP2 in Cd‐induced transcellular autophagy in AML12 cells. After silencing TNFAIP2, the cells were treated with 5 µm Cd for 6 h, and the level of transcellular autophagy was analyzed using flow cytometry. As shown in Figure 7D, TNFAIP2 silencing inhibited Cd‐induced transcellular autophagy. This indicated that Cd‐induced transcellular autophagy depends on the TNFAIP2‐TNT system.

### 2.8 Protective Effect of the TNFAIP2‐TNT System‐Mediated Transcellular Autophagy on Cd‐Exposed AML12 Cells

**Figure 8:**
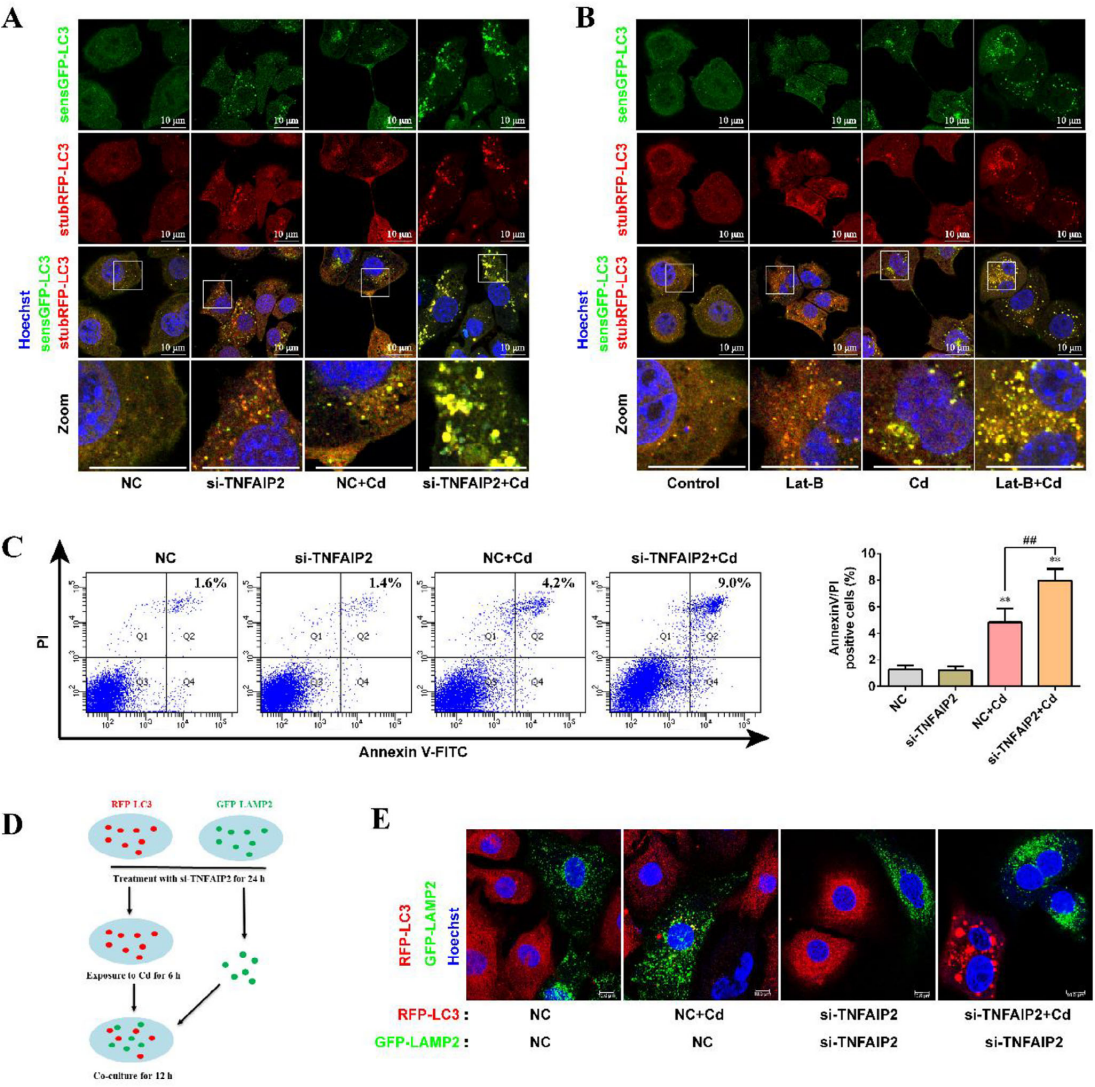
Protective effect of the TNFAIP2‐TNT system‐mediated transcellular autophagy on Cd‐exposed AML12 cells. After silencing TNFAIP2, RFP‐GFP‐LC3‐labeled AML12 cells were treated with 10 µm Cd for 6 h. A) Confocal microscopy images of RFP‐GFP‐LC3 puncta. Scale bar, 10 µm. RFP‐GFP‐LC3‐labeled AML12 cells were pretreated with 100 nm Lat‐B for 2 h and then treated with 10 µm Cd for 6 h. B) Confocal microscopy images of RFP‐GFP‐LC3 puncta. Scale bar, 10 µm. After silencing TNFAIP2, AML12 cells were treated with 10 µm Cd for 6 h. C) Flow cytometry analysis of apoptosis rate (*n* = 3). RFP‐LC3‐labeled AML12 cells (treatment with or without si‐TNFAIP2) were treated with 10 µm Cd for 6 h, and then co‐cultured with GFP‐LAMP2‐labeled AML12 cells (treatment with or without si‐TNFAIP2) for 12 h. D) Schematic diagram of co‐culture model. E) Confocal images of RFP‐LC3 puncta. Scale bar, 10 µm. Data are expressed as the mean ± SD. Statistical analysis was performed using one‐way analysis of variance and Scheffe's F test. Compared with the control group, ^*^ *p* < 0.05, ^**^ *p* < 0.01. Compared with the NC+Cd group, ^#^ *p* < 0.05, ^##^ *p* < 0.01.

We investigated the role of the TNFAIP2‐TNT system in Cd‐induced autophagic flux blockage and apoptosis in AML12 cells. We first analyzed changes in autophagic flux by silencing TNFAIP2 or using Lat‐B to inhibit the formation of TNTs. As shown in **Figure** **8**A, silencing TNFAIP2 exacerbated Cd‐induced autophagic flux blockage. Similar results were obtained when the formation of TNTs was inhibited using Lat‐B (Figure 8B). Subsequently, after silencing TNFAIP2, cells were treated with 5 µm Cd for 6 h to analyze the changes in apoptosis rate. As shown in Figure 8C, silencing TNFAIP2 exacerbated Cd‐induced apoptosis. These results indicate that the inhibiting of the TNFAIP2‐TNT system can exacerbate Cd‐induced autophagic flux blockage and apoptosis in AML12 cells.

Finally, in the co‐culture model used to evaluate the transfer of autophagosomes from injured to normal cells, we further verified the role of TNFAIP2‐TNT system‐mediated transcellular autophagy in Cd‐induced autophagic flux blockage in AML12 cells. The co‐culture model is shown in Figure 8D. Following the silencing of TNFAIP2 in RFP‐LC3‐labeled AML12 cells, the cells were exposed to 10 µm Cd for 6 h. Subsequently, GFP‐LAMP2‐labeled AML12 cells that had been silenced for TNFAIP2 were inoculated and co‐cultured for 12 h. Changes in LC3 puncta were observed using laser confocal microscopy. Figure 8E shows that in the co‐culture group without silencing TNFAIP2, the LC3 puncta of Cd‐injured cells (labeled with RFP‐LC3) were transferred to adjacent normal cells (labeled with GFP‐LAMP2) and fused with lysosomes. However, in the co‐culture group with silenced TNFAIP2, no RFP‐LC3 puncta were observed in normal cells (labeled with GFP‐LAMP2), whereas a significant accumulation of LC3 puncta was observed in Cd‐injured cells (labeled with RFP‐LC3). These findings suggest that after inhibiting of the TNFAIP2‐TNT system, autophagosomes in Cd‐injured cells could not be transferred to normal cells and accumulated. This further demonstrates that the TNFAIP2‐TNT system‐mediated transcellular autophagy is a protective mechanism for alleviating Cd‐induced autophagic flux blockage in AML12 cells.

## 3 Discussion

Autophagy is a multi‐step dynamic degradation process that maintains cellular homeostasis by degrading damaged organelles, aggregated proteins, and lipids and recycling their components.^[^ ^9^, ^45^ ^]^ It is generally accepted that the complete process of autophagy is independently completed within the same cell. However, recent studies have revealed autophagosome transfer between cells, indicating the possibility of transcellular degradation of autophagosomes.^[^ ^37^, ^42^, ^43^ ^]^ Here, we found that autophagosomes can transfer from one cell to another in a TNT‐dependent manner and fuse with lysosomes in adjacent cells for degradation. This is not a simple phenomenon of autophagosome transfer, but a regulated and complete autophagy process called transcellular autophagy (Figure S5, Supporting Information). We used different fluorescent markers to distinguish between receptor and donor cells, and successfully observed the complete dynamic process of transcellular autophagy for the first time through high‐content analysis system real‐time imaging. Unlike classical autophagy processes, the complete process of transcellular autophagy is not limited to a single cell but requires the transfer of autophagosomes to neighboring cells, where they ultimately fuse with lysosomes and undergo degradation. This new autophagy pathway provides cells with a new autophagosome clearance strategy to cope with the excessive accumulation of autophagosomes caused by incomplete autophagy. However, the current understanding of the mechanisms and functions of transcellular autophagy remains limited. This study focused on the directionality and pathways of autophagosome transfer during transcellular autophagy, as well as the conditions and upstream signals that trigger transcellular autophagy activation. We also explored the functions of transcellular autophagy.

We explored the directionality of autophagosome transfer during Cd‐induced transcellular autophagy. However, the direction of organelle transfer remains unclear. Mitochondria are currently substantial interest in the study of organelle transfer. Intercellular mitochondrial transfer is bidirectional.^[^ ^41^, ^46^ ^]^ Notably, most recent studies indicate that mitochondria tend to be preferentially transferred from healthy to damaged cells. For example, in most studies using MSCs as donor cells, as a rescue mechanism, MSCs transfer mitochondria to damaged cells through TNTs to enhance the mitochondrial function and energy metabolism of damaged cells, and even rescue damaged cells.^[^ ^47^, ^48^, ^49^, ^50^, ^51^ ^]^ Although autophagosome transfer has been reported, evidence supporting its directionality remains scarce. Our study found that intercellular autophagosome transfer is also bidirectional, but tends to transfer from Cd‐injured cells to healthy cells. Nevertheless, the differences in the status of autophagosomes that are transferred in different directions and the regulatory mechanisms governing their directional transfer are unclear.

Next, we explored the autophagosome transfer pathways during Cd‐induced transcellular autophagy. The transfer of mitochondria between cells can be achieved through various ways, including TNT, extracellular vesicles, and gap junction channels (GJCs).^[^ ^52^ ^]^ Although reports on autophagosome transfer suggest that TNTs play a significant role, there is currently insufficient evidence to indicate whether other pathways are involved. In the present study, we ruled out the importance of extracellular vesicles/endocytosis and GJCs in Cd‐induced autophagosome transfer. Instead, we confirmed that TNT is the primary pathway of autophagosome transfer during Cd‐induced transcellular autophagy. Notably, our TEM analysis revealed the presence of autophagosomes within TNTs, providing intuitive and compelling evidence for TNT‐mediated autophagosome transfer.

The mechanism of TNT formation is highly complex, and there is still a lack of comprehensive understanding of the regulatory mechanisms involved in its formation process. Despite this, several proteins have been identified in different cell types that participate in the regulation of TNT formation, including TNFAIP2, RHOC, MYO10, RALBP1, LST1, CDC42, RHOA, RHOB, S100A4, and GJA1.^[^ ^28^, ^53^, ^54^, ^55^ ^]^ However, the key proteins regulating TNT formation may vary among different cell types, and the same protein may have opposite effects on TNT formation in different cell types.^[^ ^56^ ^]^ This suggests the existence of cell‐specific mechanisms underlying TNT formation. TNFAIP2 was one of the earliest markers identified in of TNT formation, and its crucial role has been confirmed in multiple cell types.^[^ ^57^, ^58^ ^]^ In our study, among the validated proteins, only TNFAIP2 inhibited Cd‐induced TNT formation in AML12 cells. Furthermore, after silencing TNFAIP2, Cd‐induced autophagosome transfer was almost completely inhibited. These results emphasize the crucial role of the TNFAIP2‐TNT system in Cd‐induced transcellular autophagy.

Next, we explored the conditions required for the activation of transcellular autophagy. We found that incomplete autophagy and the formation are prerequisites for transcellular autophagy activation. Previous studies have shown that starvation activates autophagy and induces the generation of TNTs.^[^ ^59^, ^60^ ^]^ Our findings revealed that starvation alone was insufficient to directly trigger transcellular autophagy. Starvation‐induced autophagy is complete autophagy with intact fusion and degradation mechanisms, allowing autophagosomes to be efficiently cleared through autophagic pathways.^[^ ^59^ ^]^ Conversely, Cd‐induced autophagy is incomplete, with the disruption of autophagic fusion mechanisms, resulting in the accumulation of autophagosomes that cannot be cleared.^[^ ^44^ ^]^ Therefore, we speculate that the trigger of transcellular autophagy may be related to the disruption of autophagic fusion mechanisms. Our results support this hypothesis. We found that, while activating autophagy and inducing the formation of TNTs, blocking the fusion of autophagosomes and lysosomes activated transcellular autophagy. In summary, our findings revealed that incomplete autophagy and the formation of TNTs are necessary conditions for transcellular autophagy.

After identifying the conditions necessary for transcellular autophagy activation, we explored the upstream signals that trigger this activation. ROS are upstream signals for autophagy activation and TNT formation, and excessive accumulation of ROS can lead to autophagic flux blockage.^[^ ^61^, ^62^, ^63^ ^]^ Autophagy, TNT formation, and disruption of the autophagy fusion mechanism (which can lead to autophagic flux blockade) are necessary conditions for transcellular autophagy activation. Additionally, ROS plays a pivotal role as an upstream signal in Cd‐induced cell injury.^[^ ^15^ ^]^ Therefore, this study focused on the role of ROS in transcellular autophagy activation. Our study revealed that clearing ROS accumulation using NAC completely inhibited Cd‐induced transcellular autophagy. Additionally, ROS clearance inhibited Cd‐induced autophagy activation and TNT formation, suggesting that ROS accumulation is a vital upstream signal for Cd‐induced transcellular autophagy. Notably, we discovered two drugs, H_2_O_2_ and DMNQ, that activate transcellular autophagy in AML12 cells. DMNQ, a ROS inducer, can trigger ROS‐dependent cell death.^[^ ^64^ ^]^ Our findings show that inducing ROS accumulation through H_2_O_2_ or DMNQ can promote TNT generation and induce autophagic flux blockade, ultimately triggering transcellular autophagy. This further confirms that ROS accumulation is an important driving factor of transcellular autophagy.

Autophagy is a vital cellular self‐cleaning process that plays a crucial role in the maintenance of cellular homeostasis and survival.^[^ ^65^ ^]^ Incomplete autophagy refers to a malfunctioning process in which accumulated autophagosomes fail to fuse with lysosomes for degradation. This ultimately leads to blockage of autophagic flux and excessive accumulation of autophagosomes, which are often detrimental to cell survival.^[^ ^66^ ^]^ A recent study revealed that healthy and AGE‐damaged podocytes can exchange lysosomes and autophagosomes through TNTs. This exchange mechanism helps to alleviate lysosomal dysfunction, excessive accumulation of autophagosomes, and apoptosis in damaged cells. However, this protective effect was eliminated by the tnfaip2 deletion or TNT inhibition.^[^ ^37^ ^]^ This suggests that the transfer of autophagosomes to neighboring cells may be a protective mechanism against excessive accumulation of autophagosomes. In this study, we found that silencing TNFAIP2 or inhibiting the formation of TNTs exacerbated Cd‐induced autophagic flux blockage and apoptosis. Furthermore, in co‐culture models, we found that Cd‐injured cells could transfer autophagosomes to normal cells for degradation, which helped alleviate the accumulation of autophagosomes in Cd‐injured cells. However, upon silencing TNFAIP2, autophagosomes in Cd‐injured cells failed to be transferred to normal cells, leading to their accumulation. These findings suggest that the TNTAIP2‐TNT system‐mediated transcellular autophagy is a protective mechanism that alleviates the excessive accumulation of autophagosomes induced by incomplete autophagy.

The current study has some limitations. First, it lacks validation in other hepatocyte models, particularly primary hepatocytes. However, technical challenges in subculturing and genetic manipulation of primary hepatocytes restricted further verification in this study. Future research will explore improved methodologies for conducting such studies in primary hepatocytes or perform validation across different cell lineages. Second, the experiment was conducted under the traditional 2D culture conditions. In contrast, 3D spheroid cultures mimic the in vivo cellular microenvironment more closely. Therefore, future research will consider applying 3D spheroid culture techniques to better reflect cell–cell interactions. Third, owing to the numerous technical challenges in in vivo research, such as difficulties in multiple fluorescent labeling of live cells, accurate differentiation between damaged and healthy cells, and real‐time monitoring, the results of this study have not yet been verified in vivo. Therefore, future research should consider the differences in the physiological environments between in vivo and in vitro studies to design a more scientific in vivo experimental protocol.

In summary, we revealed a novel autophagy pathway, transcellular autophagy, which serves as a protective mechanism for cells to mitigate the excessive accumulation of autophagosomes caused by incomplete autophagy. Transcellular autophagy has substantial research potential in diseases associated with autophagy dysfunction. Our study provides novel insights and directions for strategies targeting autophagy for disease intervention.

## 4 Experimental Section

### Reagents and Antibodies

Cd chloride (CdCl_2_, 202908), dexamethasone (D4902), Hoechst33258 (94403), Lucifer Yellow CH dilithium salt (LY, L0259), and Rhodamine B isothiocyanate‐Dextran (RD, R9379) were purchased from Sigma–Aldrich (St. Louis, MO, USA). Dulbecco's modified Eagle's Medium/Nutrient Mixture F‐12 (D‐MEM/F12, 12400024), fetal bovine serum (FBS, 10437028), and insulin‐transferrin‐selenium‐A supplement (ITS‐A, 51300044) were purchased from Gibco (Grand Island, NY, USA). RAPA (HY‐10219),3‐MA (HY‐19312), TG (HY‐13433), Lat‐B (HY‐101848), Gap 27 (HY‐P0139), GA (HY‐N0180), Compound C (HY‐13418A), and DMNQ (HY‐121026) were purchased from MedChem Express (Monmouth Junction, NJ, USA). Hoechst 33342 (C1028), EBSS (C0214), and NAC (S0077) were purchased from Beyotime (Shanghai, China). The Annexin V‐fluorescein isothiocyanate (FITC)/propidium iodide (PI) Apoptosis Detection Kit (A211) was purchased from Vazyme (Nanjing, China). TRITC Phalloidin (CA1610) and ROS assay kits (CA1410) were purchased from Solarbio (Beijing, China). The Polyplus‐transfection reagent was purchased from Polyplus‐transfection (Illkirch, France). Anti‐LST1 (ab252839, 1:2000), Anti‐CDC42 antibody (ab187643, 1:10 000), and Anti‐TNFAIP2 (ab196659, 1:1000) antibodies were purchased from Abcam (Cambridge, UK). Anti‐RHOC (PS02067, 1:1000), Anti‐MYO10 antibody (PS02569, 1:1000), and Anti‐RALBP1 (T55676, 1:1000) antibodies were purchased from Abmart (Shanghai, China).

### Cell Culture and Treatment

The mouse hepatocyte cell line, AML12 (ATCC CRL‐2254, Manassas, VA, USA) was seeded in D‐MEM/F12 medium supplemented with 10% FBS, 1% ITS‐A, and 40 ng mL^−1^ dexamethasone. Cells were incubated in a constant‐temperature incubator at 37 °C with 5% CO_2_ and saturated humidity. A serum‐free medium was used for Cd exposure.

### Lentivirus (LV) Infection and siRNA Transfection

AML12 cells were seeded in a 24‐well plate and cultured to a density of ≈20%. Following the manufacturer's protocol, the lentivirus was incubated with the cells for 48–72 h. Subsequently, the cells were cultured in complete medium containing 4 µg mL^−1^ of puromycin to select positive cells and establish stable cell lines. RFP‐LC3, stubRFP‐sensGFP‐LC3, GFP, and GFP‐LAMP2 lentiviruses (GeneChem Corporation, Shanghai, China) were used. For siRNA transfection, siRNA was mixed with the Polyplus transfection reagent according to the manufacturer's protocol. The mixture was then added to fresh complete medium without penicillin or streptomycin, and the cells were transfected for 24 h. Silencing efficiency was analyzed using WB. The siRNA sequences used in this study were purchased from RiboBio Corporation (Guangzhou, China) and are listed in Table S1 (Suppoerting Information).

### High‐Content Analysis System

RFP‐LC3‐ and GFP‐labeled AML12 cells were cultured separately or mixed in 96‐well culture plates. After reaching 70–80% confluence, the cells were treated with 5 µm Cd and labeled with Hoechst 33 342 to label the cell nucleus. Images were captured every 2 min using a high‐content analysis system (PerkinElmer Company, USA).

### Confocal Fluorescence Analysis

RFP‐LC3‐labeled cells were cultured either alone or mixed with GFP‐ or GFP‐LAMP2‐labeled cells in a 1:1 ratio on a 24‐well glass slide. The cells were treated according to the experimental requirements. After treatment, the cells were fixed with 4% paraformaldehyde, stained with Hoechst 33 258 to label the cell nuclei, and imaged using a laser confocal microscope.

### Analysis of Flow Cytometry

For the analysis of intercellular autophagosome transfer in rat, RFP‐LC3‐labeled cells were co‐cultured with GFP‐LC3‐labeled cells at a ratio of 1:1 and treated according to experimental requirements. Following treatment, the cells were collected and analyzed for the double‐positive fluorescence rate using a FACS LSRFortessa flow cytometer (BD Company, USA).

For the analysis of ROS levels, the cells were treated according to the experimental requirements, collected through trypsin digestion, and incubated with the DCFH‐DA working solution at 37 °C for 20 min in the dark. During incubation, the solution was mixed and inverted every 3–5 min to ensure uniform exposure. Subsequently, the residual working solution was washed three times with serum‐free medium to remove the unreacted reagents. Finally, the ROS levels in the cells were analyzed using flow cytometry.

For apoptosis rate analysis, after treatment, cells were collected through trypsin digestion (without EDTA). Then, 5 µL of Annexin V‐FITC and PI were added in sequence, and the cells were incubated at room‐temperature for 20 min in the dark. The rate of apoptosis was analyzed using flow cytometry.

### Cell Co‐Culture Models

For the Transwell co‐culture model, the Transwell chamber (0.4 µm‐aperture) in a six‐well plate was placed upside down in a 50 mL beaker. RFP‐LC3‐labeled cells were then inoculated into the outer chamber of a Transwell plate and cultured for 12 h. Simultaneously, the GFP‐LC3‐labeled cells were inoculated into a six‐well plate and cultured for 12 h. The Transwell chamber was transferred to a six‐well plate and co‐cultured with GFP‐LC3‐labeled AML12 cells. After treatment with 10 µm Cd for 6 h, the GFP‐LC3‐labeled AML12 cells was collected from the six‐well plate using trypsin digestion and the fluorescence double‐positive rate was analyzed using flow cytometry.

To evaluate the transfer of autophagosomes from normal to injured cells, GFP‐labeled cells were cultured in a six‐well plate. Following treatment with 10 µm Cd for 6 h, the culture medium was discarded and replaced with complete medium containing RFP‐LC3‐labeled cells. After co‐culturing for 12 h, the double‐positive fluorescence rate was analyzed using flow cytometry.

To evaluate the transfer of autophagosomes from injured to normal cells in the co‐culture model, RFP‐LC3‐labeled cells were cultured in six‐well plates. Following treatment with 10 µm Cd for 6 h, the culture medium was discarded and replaced with complete medium containing GFP‐labeled cells. After co‐culturing for 12 h, the double‐positive fluorescence rate was analyzed using flow cytometry.

### Phalloidin Staining

After treatment, the cells were fixed with 4% formaldehyde solution at roommtemperature for 10 min, permeabilized with 0.5% Triton X‐100 solution for 5 min, incubated with phalloidin working solution at room‐temperature in the dark for 30 min, labeled with Hoechst 333 258 to mark the cell nucleus, and imaged using a confocal microscope (TCS SP8 STED, Leica, Germany) or an inverted fluorescence microscope (DMI3000B, Leica, Germany).

### TEM Analysis

After exposure to 10 µm Cd for 6 h, the cells were sequentially fixed with 2.5% glutaraldehyde and 1% osmium tetroxide. The samples were then dehydrated in ethanol and acetone. After immersion in acetone, resin embedding, and polymerization, ultrathin sections were prepared. The samples were then stained with uranium acetate and lead citrate before being photographed using TEM (HT7800 model, Hitachi High‐Tech, Tokyo, Japan).

### Scrape Loading/Dye Transfer (SL/DT) Analysis

The SL/DT analysis was conducted in accordance with the previous study to evaluate gap junction intercellular communication.^[^ ^67^ ^]^ After treatment, cell scratches were made using surgical blades, incubated at 37 °C for 5 min with PBS containing LY (0.5 mg mL^−1^) and RD (2.5 mg mL^−1^), and then fixed with 4% paraformaldehyde. Finally, the fluorescent diffusion distance from the scraped edge to the adjacent cells was measured using a fluorescence microscope (DMI3000B, Leica, Germany).

### RNA‐seq Analysis

The cells were treated with 10 µm Cd for 6 h, and total RNA was extracted. RNA‐seq was performed by BGI Genomics Co Ltd. (Shenzhen, China). The raw data obtained from sequencing were filtered using SOAPnukev1.5.6 to obtain clean data. HISAT2 v2.1.0 and Bowtie2 v2.3.4.3 software were used to align the clean data against the reference genome sequence, while RSEM v1.3.1 software was used for gene expression quantification. Pheatmapv1.0.8 was used to draw a cluster heatmap of gene expression in different samples. DEGs were identified using a Q‐value ≤ 0.05 as the threshold and Phyper was applied for KEGG enrichment analysis of DEGs.

### WB

Cells were collected using RIPA lysis buffer supplemented with 1% protease inhibitors. The protein concentration of the sample was determined using the BCA colorimetry and lysis buffer was added to standardize the protein concentration. Subsequently, SDS loading buffer was added and denatured to obtain a protein sample. Following SDS‐PAGE, the proteins were transferred onto polyvinylidene fluoride membranes. The membranes were then sequentially incubated with the corresponding primary and secondary antibodies. Target proteins were visualized using the ECL chemiluminescence. The grayscale value was analyzed using ImageJ software (National Institutes of Health, United States) and the ratio of the grayscale value of the target protein to that of β‐actin was used to indicate the relative expression level of the target protein.

### Statistical Analysis

Data from at least three independent experiments were analyzed one‐way analysis of variance and Scheffe's F test using GraphPad Prism 6 software (GraphPad Software Inc., La Jolla, CA, USA). The results are shown as the mean ± SD, with *p* > 0.05 considered not statistically significant and *p* < 0.05 or *p* < 0.01 considered statistically significant.

## Conflict of Interest

The authors declare no conflict of interest.

## Supporting information



Supporting Information

Supplemental Video 1

Supplemental Video 2

## Data Availability

The data that support the findings of this study are available in the supplementary material of this article.;
