# Supplementary material for: Tunneling Nanotube‐Mediated Transcellular Autophagy Alleviates Cadmium Induced Hepatocyte Injury
Source: Adv Sci (Weinh). 2025 Jul 28;12(37):e02793. doi: 10.1002/advs.202502793 (PMC12499425; doi:10.1002/advs.202502793)
Supplement: Supplementary file 1 — Supporting Information [file ADVS-12-e02793-s003.docx]

Supporting Information

Tunneling Nanotube-Mediated Transcellular Autophagy Alleviates Cadmium Induced Hepatocyte Injury

*Tao Wang, Li Wang, Jian Sun, Yan Chen, Waseem Ali, Yonggang Ma, Ruilong Song, Xishuai Tong, Jiaqiao Zhu, Yan Yuan, Jianhong Gu, Jianchun Bian, Zongping Liu*, and Hui Zou**


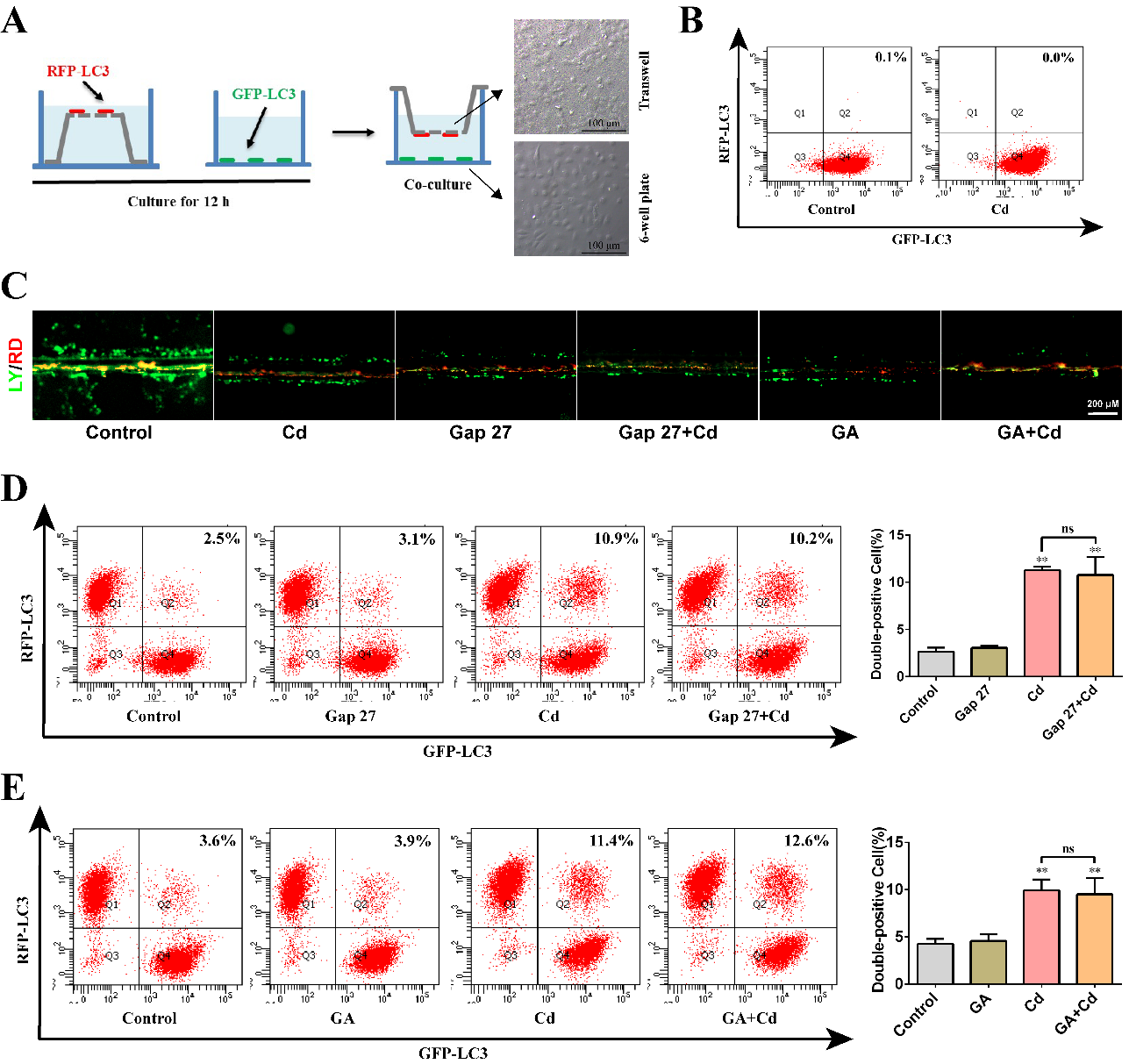


**Figure S1.** **Effects of extracellular vesicles/endocytosis and gap junction on** **Cd-induced transcellular autophagy in AML12 cells.** The Transwell chamber of the six-well plate was inverted into a 50-mL beaker. Subsequently, the RFP-LC3-labeled cells were seeded into the outer chamber of the Transwell and cultured for 12 h. Meanwhile, the GFP-LC3-labeled cells were seeded into the six-well plate and cultured for 12 h. Afterward, the Transwell chamber was transferred to the six-well plate and co-cultured with the GFP-LC3-labeled AML12 cells. A) Schematic diagram of Transwell co-culture model. B) In the Transwell co-culture model, cells were treated with 10 μM Cd for 6 h. Following the treatment, the cells in the six-well plate were collected and the LC3 transfer rate was analyzed using flow cytometry. AML12 cells were pretreated with 500 μM Gap 27 or 5 μM GA for 1 h, followed by treatment with 10 μM Cd for 6 h. C) The gap junction intercellular communication was evaluated using the SL/DT method. D, E) The rate of LC3 transfer was analyzed using flow cytometry (*n*=3). Data are expressed as the mean ± SD. Statistical analysis was performed using one-way analysis of variance and Scheffe’s F test. Compared with the control group, * *p* < 0.05, ** *p* < 0.01. Compared with the Cd group, # *p* < 0.05, ## *p* < 0.01.

**
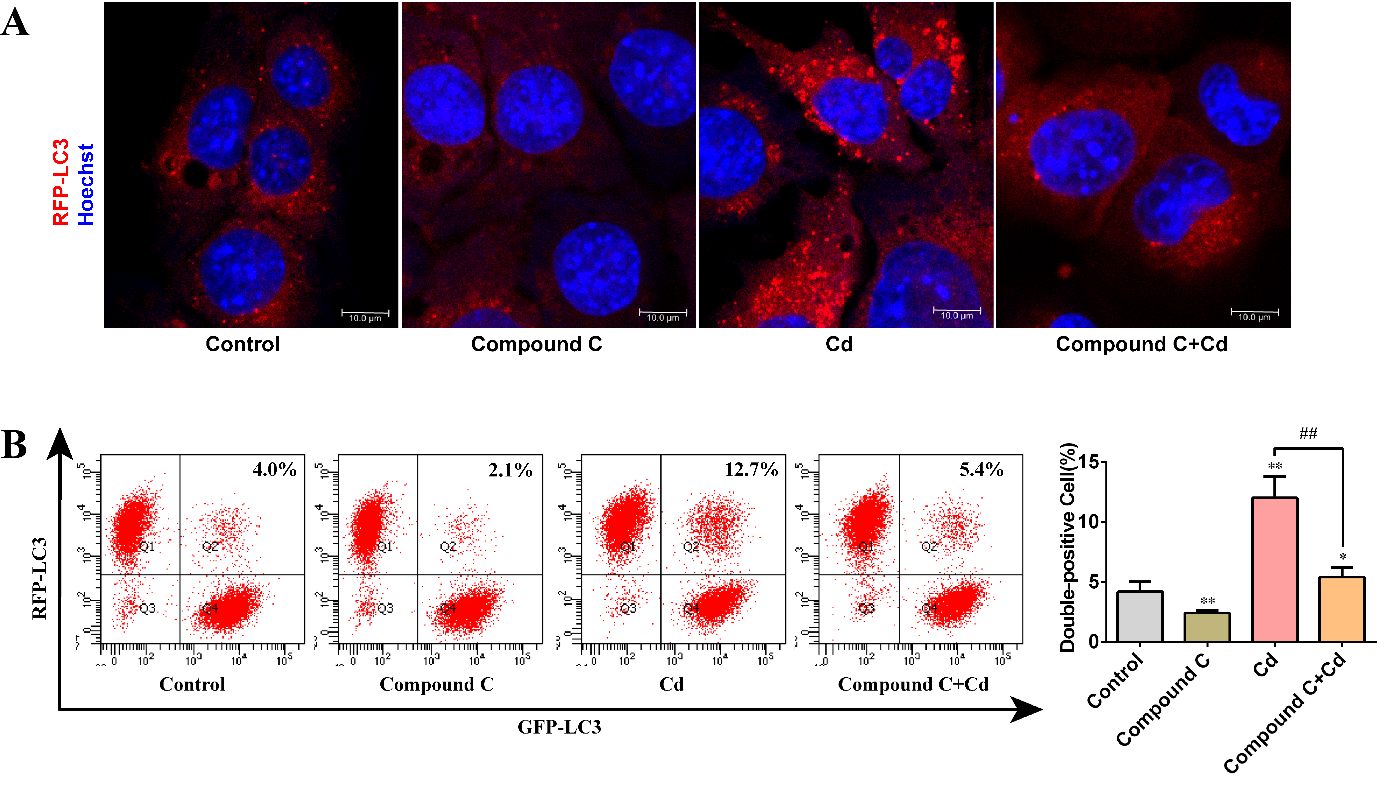
**

**Figure S2.** **Effects of inhibiting AMPK pathway on Cd-induced autophagy and transcellular autophagy.** AML12 cells were pretreated with 10 μM Compound C (the inhibitor of AMPK) for 1 h, followed by treatment with 10 μM Cd for 6 h. A) Confocal microscopy images of RFP-LC3 puncta. Scale bar, 10 μm. B) The rate of LC3 transfer was analyzed using flow cytometry (*n*=3). Data are expressed as the mean ± SD. Statistical analysis was performed using one-way analysis of variance and Scheffe’s F test. Compared with the control group, * *p* < 0.05, ** *p* < 0.01. Compared with the Cd group, # *p* < 0.05, ## *p* < 0.01.


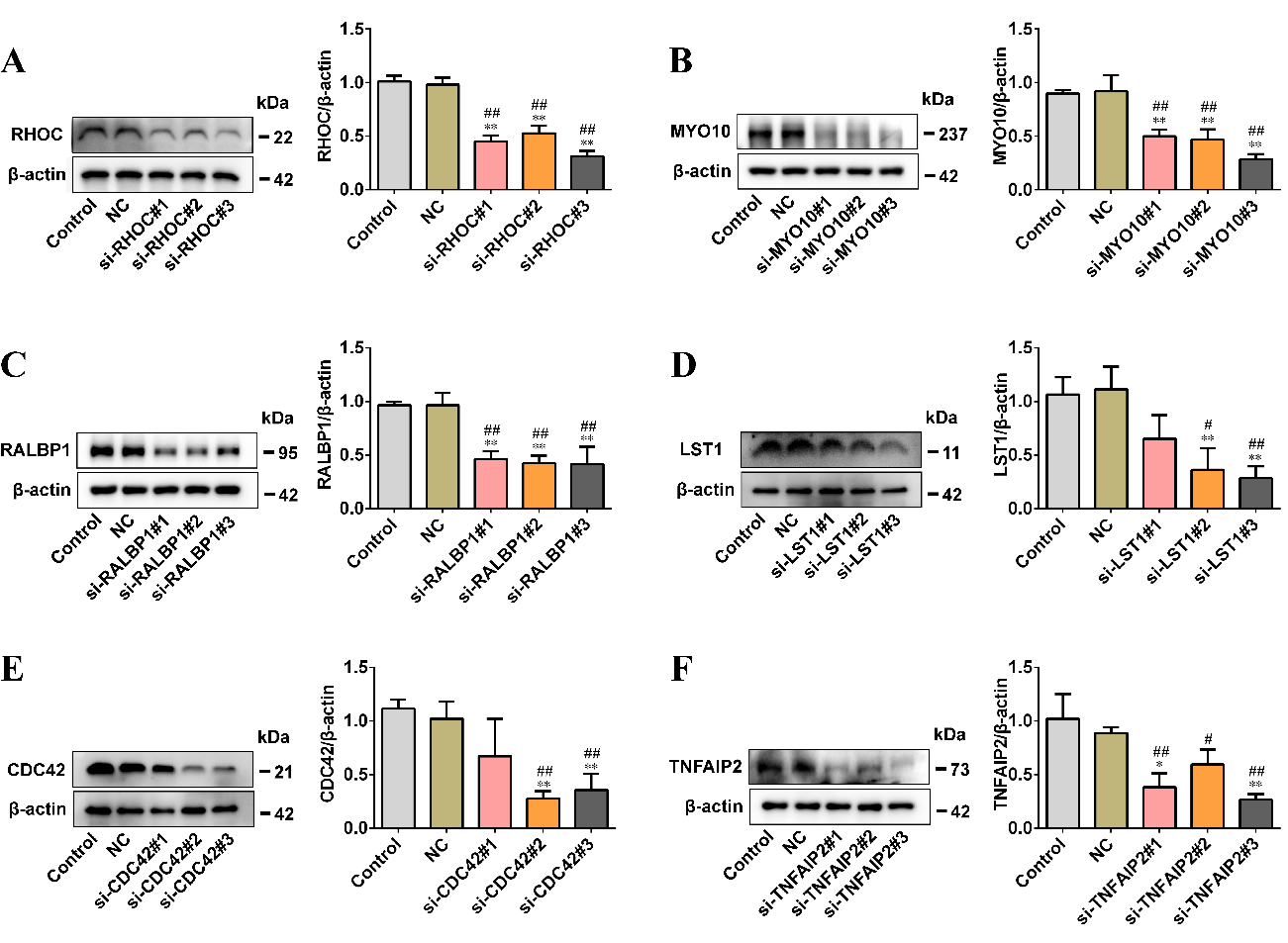


**Figure S****3.** **Verification of the silencing efficiency of TNT formation related proteins.** A–F) AML12 cells were treated with 60 nM si-RHOC, 60 nM si-MYO10, 60 nM si-RALBP1, 40 nM si-LST1, 40 nM si-CDC42, 60 nM si-TNFAIP2, or negative control (NC) siRNA for 24 h, and the protein expression levels were analyzed using WB. Data are expressed as the mean ± SD (*n* = 3). Statistical analysis was performed using one-way analysis of variance and Scheffe’s F test. Compared with the control group, * *p* < 0.05, ** *p* < 0.01. Compared with the NC group, # *p* < 0.05, ## *p* < 0.01. The si-RHOC#3, si-MYO10#3, si-RALBP1#1, si-LST1#3, si-CDC42#2, and si-TNFAIP2#3 were finally selected for this study.


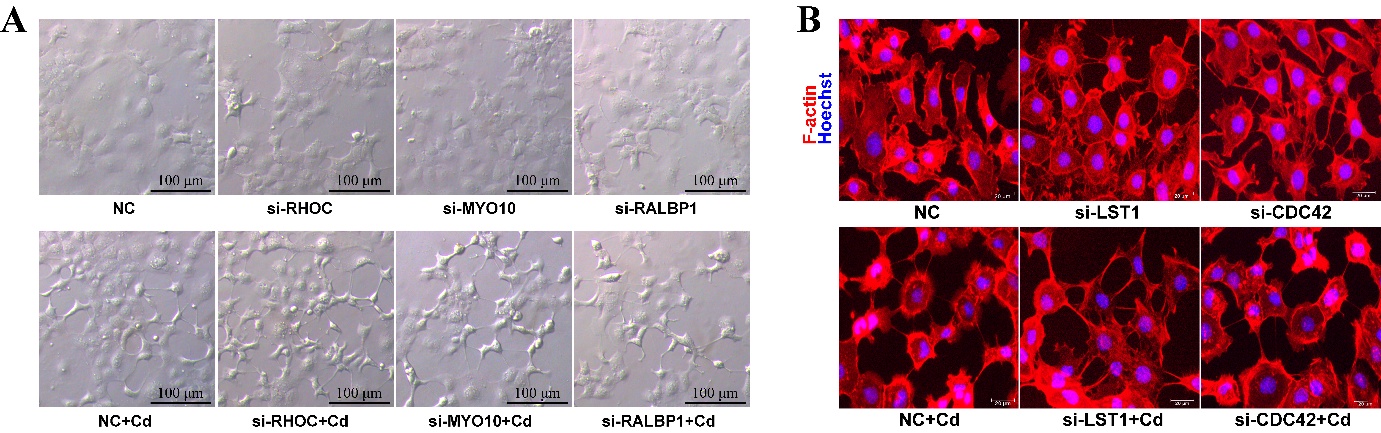


**Figure S4. Effect of silencing TNT formation related proteins on the formation of TNTs induced by Cd in AML12 cells.** A) After silencing RHOC, MYO10, or RALBP1, cells were treated with 10 μM Cd for 6 h and then the formation of TNTs was observed under brightfield microscopy. Scale bar, 100 μm. B) After silencing LST1 or CDC42, cells were treated with 10 μM Cd for 6 h and then the formation of TNTs was observed using phalloidin staining. Scale bar, 20 μm.


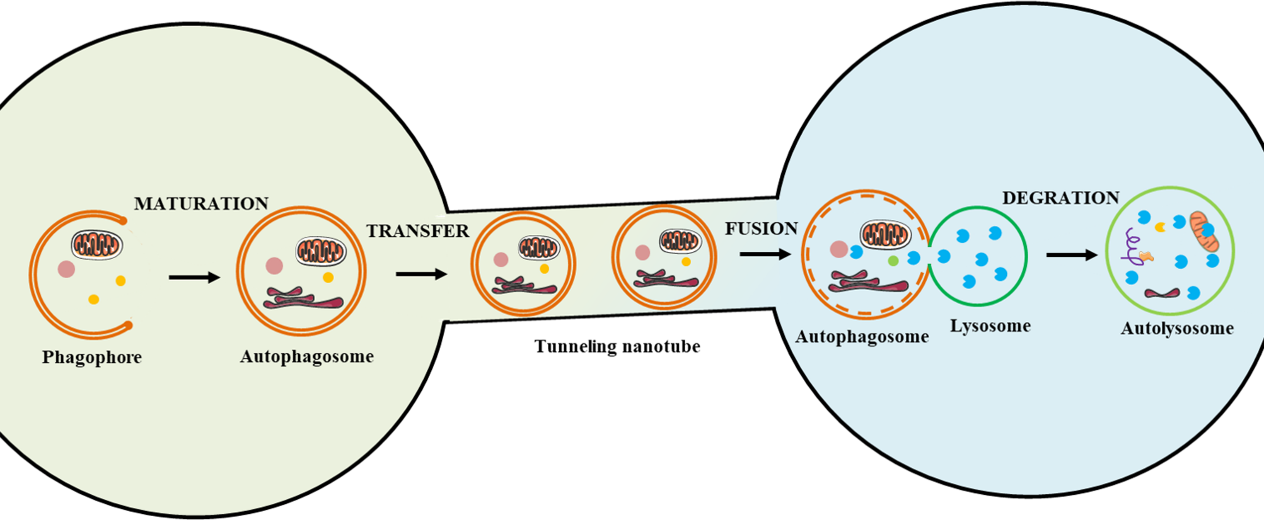


**Figure S5. Schematic diagram of transcellular autophagy.** After autophagosomes maturation, they transfer from one cell to an adjacent cell in a TNT-dependent manner and fuse with lysosomes in the adjacent cell for degradation.

Supporting Information

Tunneling Nanotube-Mediated Transcellular Autophagy Alleviates Cadmium Induced Hepatocyte Injury

*Tao Wang, Li Wang, Jian Sun, Yan Chen, Waseem Ali, Yonggang Ma, Ruilong Song, Xishuai Tong, Jiaqiao Zhu, Yan Yuan, Jianhong Gu, Jianchun Bian, Zongping Liu*, and Hui Zou**

**Table S1.** **siRNA target sequence in this study.**

| siRNA | siRNA target sequence |
| --- | --- |
| si-RHOC#1 | 5′- AGAACTATATAGCCGACAT -3′ |
| si-RHOC#2 | 5′- TACAGGTCCGGAAGAATAA -3′ |
| si-RHOC#3 | 5′- AGCUGGCCAAGAUGAAACA -3′ |
| si-MYO10#1 | 5′- GCACGAAAGCAATATAGAA -3′ |
| si-MYO10#2 | 5′- CGTTAGAAGTCTTCCAGTA -3′ |
| si-MYO10#3 | 5′- GCATTAGTCAGTCGACCAA -3′ |
| si-RALBP1#1 | 5′- GAAGAATATGAGCCTAACA -3′ |
| si-RALBP1#2 | 5′- CTACAAAGTTCCCTGGATT -3′ |
| si-RALBP1#3 | 5′- CCTGAAGCAAGTAACAAGA -3′ |
| si-LST1#1 | 5′- GTCCAGTAGTGATATCACA -3′ |
| si-LST1#2 | 5′- GTCATCATCCTGTTCATCT -3′ |
| si-LST1#3 | 5′- GTCACACTATGGTCATCTA -3′ |
| si-CDC42#1 | 5′- CCATCGGAATATGTACCAA -3′ |
| si-CDC42#2 | 5′- GGTCTCTCCATCCTCATTT -3′ |
| si-CDC42#3 | 5′- GCAAGAGGATTATGACAGA -3′ |
| si-TNFAIP2#1 | 5′- GCAAACTCCTGAGGAATTA -3′ |
| si-TNFAIP2#2 | 5′- GCACCTGCACCTAGTGAAA -3′ |
| si-TNFAIP2#3 | 5′- GGAACATACTGGACATCAA -3′ |
| si-VPS41 | 5′- GCACAATACTGCAACATCT -3′ |
